# Platelet-Rich Plasma Promotes the Proliferation of Human Keratinocytes via a Progression of the Cell Cycle. A Role of Prolidase

**DOI:** 10.3390/ijms22020936

**Published:** 2021-01-19

**Authors:** Magdalena Misiura, Tomasz Guszczyn, Ilona Oscilowska, Weronika Baszanowska, Jerzy Palka, Wojciech Miltyk

**Affiliations:** 1 Department of Analysis and Bioanalysis of Medicines, Medical University of Bialystok, Kilińskiego 1, 15-089 Bialystok, Poland; magdalena.misiura@umb.edu.pl; 2 Department of Pediatric Orthopaedics and Traumatology, Medical University of Bialystok, Kilińskiego 1, 15-089 Bialystok, Poland; tombial@me.com; 3 Department of Medicinal Chemistry, Medical University of Bialystok, Kilińskiego 1, 15-089 Bialystok, Poland; ilona.zareba@gmail.com (I.O.); w.baszanowska22@wp.pl (W.B.); pal@umb.edu.pl (J.P.)

**Keywords:** PRP, PEPD, prolidase, keratinocytes, HaCaT, proliferation, cell cycle

## Abstract

Although the role of platelet-rich plasma (PRP) in tissue regeneration has been confirmed in many studies, the mechanism of this process is still not fully understood. Human keratinocytes (HaCaT) cells were used as an experimental model for studies on the effects of PRP on cell proliferation, migration, collagen biosynthesis, prolidase activity, and its expression and anabolic signaling. The activation of epidermal growth factor receptor (EGFR), β_1_-integrin, and insulin-like growth factor-1 receptor (IGF-1R) by PRP were investigated by western blot and immunocytochemistry. It has been found that PRP induced keratinocytes migration and proliferation through activation of cell cycle progression and EGFR downstream signaling. Similar biological effects were achieved by an addition to the culture medium of prolidase (PEPD), a ligand of EGFR (PRP is a rich source of PEPD–2 ng/mL). PRP-dependent stimulation of collagen biosynthesis was accompanied by an increase in the expression of NF-κβ, IGF-1R-downstream signaling proteins, and PEPD activity. The data suggest that PRP activates a complex of growth factors and adhesion receptors that stimulate cell proliferation, migration, and collagen biosynthesis. PRP induces PEPD-dependent human keratinocyte proliferation through activation of the EGFR receptor. Our study provides a novel mechanism of PRP-dependent wound healing.

## 1. Introduction

A sample of blood rich in platelets (PLT), known also as thrombocytes, the concentration of which is above whole blood baseline value is defined as platelet-rich plasma (PRP). It is obtained by centrifugation of the blood sample and separation of the relevant blood fraction [1]. Notably, platelets contain more than 300 biologically active molecules that are released upon activation from α-granules and dense granules of platelets and subsequently may regulate the tissue regeneration process [2,3,4]. Activated thrombocytes are a source of multiple growth factors (GFs) and cytokines, including platelet-derived growth factor (PDGF), basic fibroblast growth factor (bFGF), vascular endothelial growth factor (VEGF), insulin-like growth factor-1 (IGF-1), transforming growth factor-β (TGF-β), and others [5,6]. GFs are involved in specific biomolecular functions during tissue repair playing a fundamental role in wound healing. When secreted, GFs bind to the transmembrane receptors of the target cells, inducing cell proliferation, migration, and extracellular matrix (ECM) formation, contributing to tissue repair [7,8]. The role of PRP in tissue regeneration and wound healing has been confirmed in many studies. However, the mechanism of this process is still not fully understood. Some findings suggest that PRP has a strong effect on vascularization. PRP contains VEGF that promotes the formation of vessels in burn wounds [5,9]. Therefore, PRP is used in clinical treatment of acute and chronic wounds with good results. PRP can also increase the healing rate of diabetic wounds, reduce the injured area of ulcers, and improve joint function in osteoarthritis [5,10,11,12]. Recent studies suggest that prolidase could play an important role in the tissue repair process [13]. 

Prolidase [E.C.3.4.13.9] known also as Peptidase D (PEPD) or imidopeptidase, is a cytoplasmic enzyme, which cleaves dipeptides or tripeptides [1] with C-terminal proline or hydroxyproline [2]. The enzyme activity is stimulated by the signaling of integrins, IGF-1, and TGF-β receptors, which also stimulate collagen biosynthesis [14,15]. An increase in prolidase activity supports proline availability for collagen biosynthesis. Of special interest is the ability of extracellular PEPD to interact directly with the epidermal growth factor receptor (EGFR), inducing EGFR-dependent signaling [16]. Moreover, recent reports have indicated that PEPD can bind p53, protecting its translocation to the nucleus [17]. Regarding the tissue specificity of prolidase, the highest level of prolidase mRNA expression is observed in the kidneys, small intestine, and duodenum [18], while high prolidase activity has been reported in erythrocytes and human skin fibroblasts [19]. Guszczyn et al. [1] showed that PRP is an important source of prolidase [16], however, its role in wound healing is not known. Since prolidase evokes several anabolic activities, we hypothesized that it is an important player in the mechanism of the PRP-dependent wound healing process in an experimental model of human keratinocytes.

## 2. Results

### 2.1. PRP Promotes DNA Biosynthesis and Cell Proliferation/Migration of HaCaT Keratinocytes

**Figure 1:**
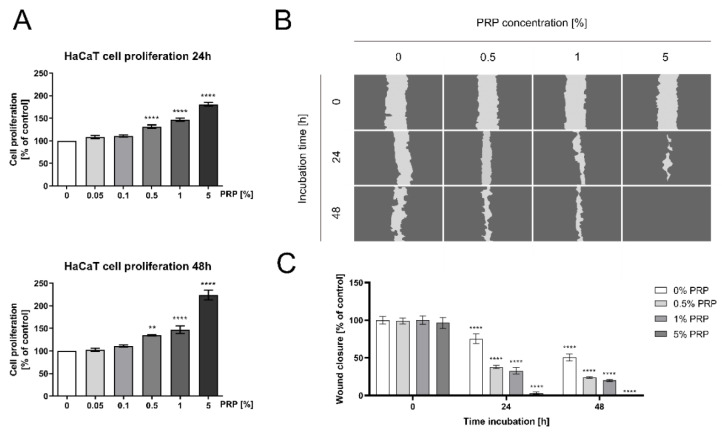
Platelet-rich plasma (PRP)-dependent stimulation of DNA biosynthesis and cell migration of human keratinocytes (HaCaT). (**A**) HaCaT cell proliferation after 24 and 48h incubation with PRP; (**B**) HaCaT cell migration in a model of wound healing/scratch assay; (**C**) wound healing rate of HaCaT cells. Statistical significances were expressed using asterisks such as, ** <0.01, and **** <0.0001.

Since previous studies suggested the possible effect of PRP on enhanced re-epithelization of keratinocytes during wound healing [20], the cell monolayer was mechanically scratched and treated with different PRP concentrations (0.05%, 0.1%, 0.5%, 1%, and 5%). After 24 and 48 h incubation, the DNA biosynthesis and cell migration were measured. The possible effect of PRP on the enhanced proliferation of keratinocytes was studied in an experimental model of wound healing [20]. As shown in Figure 1A, PRP stimulated DNA synthesis in a dose- and time-dependent manner. The 5% concentration of PRP strongly stimulated this process after 24 and 48 h incubation (180.932 ± 8.979% and 223.762 ± 24.496% of control, respectively). The results demonstrated that cell proliferation was accelerated under PRP treatment. We also observed improved viability of PRP-treated human keratinocytes (Figure S1). Based on these results further investigations were conducted on PRP at concentrations of 0.5–5%.

The effect of PRP on the migration of keratinocytes was subsequently evaluated using an in vitro wound closure/scratch assay. The results showed that the injury area was gradually closed in a time- and dose-dependent fashion (Figure 1B). After 48 h incubation with 5% PRP, the cells completely covered the area of the wound while the wounded area of control cells remained uncovered. The data indicate that human keratinocytes exhibit a strong capability of wound closure in an experimental model of wound healing (Figure 1C).

### 2.2. PRP Stimulates Cell Cycle Progression of HaCaT Keratinocytes

The expression of cell-cycle regulatory proteins (cyclin A2, B2, and E1), CDT1, and cell-cycle-regulated enzymes (phospho-cdc-2, thymidine kinase 1) and fixed-cell cycle analysis were studied in PRP (0.5–5%, 24h) treated keratinocytes. It was confirmed by fixed cell cycle analysis. 

**Figure 2:**
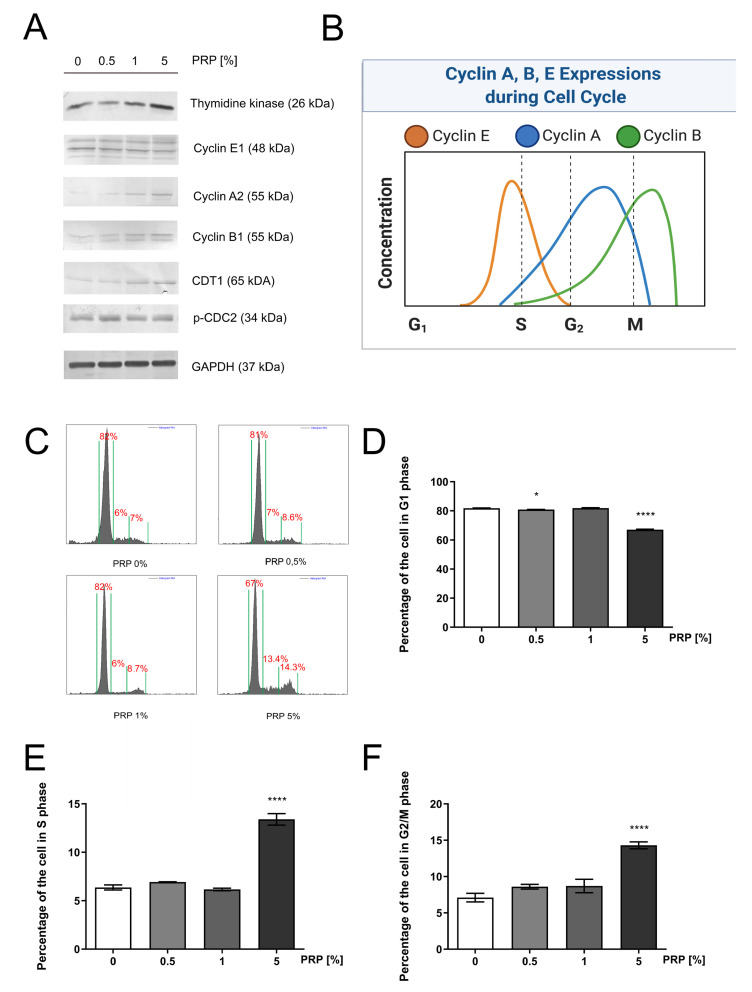
PRP-induced progression of the cell cycle of HaCaT keratinocytes (**A**) Expression of the selected cell-cycle regulatory proteins in PRP-treated keratinocytes; (**B**) Illustration of the cyclin-dependent cell cycle. Created with BioRender.com. (**C**) Ethanol-fixed cell cycle analysis of HaCaT cells after 24h incubation with PRP; (**D**) Percentage of keratinocytes in the G1 phase; (**E**) Percentage of keratinocytes in the S phase; (**F**) Percentage of keratinocytes in the G2/M phase. Statistical significances were expressed using asterisks such as * <0.05, and **** <0.0001.

Western immunoblotting analysis revealed that the expression of thymidine kinase 1 was strongly increased in PRP-treated HaCaT cells (Figure 2A). An enzyme required for deoxythymidine triphosphate (dTTP) synthesis is thymidine kinase 1; dTTP serves as a substrate for DNA biosynthesis [21]. Figure 2A demonstrates that PRP remarkably elevated the expression of cyclin A2, B2, and E1 in a dose-dependent manner. Cyclin E1 reaches the peak of its activation in the G1/S boundary, allowing the cell to enter the S-phase. CDT1 controls DNA replication during the S-phase [22] and we found that PRP induced expression of this protein in a dose-dependent manner (Figure 2A). Cyclin A2 is a well-established cell cycle regulator of the S-phase, while cyclin B2 is detectable afterward in mitosis. One of the mitosis markers is cdc2 kinase that is activated via phosphorylation at Thr^161^ [23]. This kinase was found to be activated upon PRP treatment (Figure 2A). A graphical illustration of the cyclin-dependent regulation of cell cycle presents Figure 2B.

As shown in Figure 2C, cell cycle progression increases in a PPR concentration-dependent manner. The percentage of cells in the G1-phase noticeably decreases after the PRP treatment, in a dose-dependent manner (81.850 ± 1.261%, 82.435 ± 0.403%, 72.725 ± 3.753%) for cultures treated with 0.5%, 1%, and 5% PRP respectively, versus 82.745 ± 1.493% in the control group. Compared to the control, cells subjected to PRP have significantly increased DNA synthesis (S phase) as well as a higher number of cells that entered mitosis (G2/M phase) (Figure 2D–F). These findings indicate that PRP increased cell proliferation via alterations in key cell-cycle regulatory proteins. 

### 2.3. PRP Induces EGFR and EGFR-Downstream Signaling Proteins Expression in HaCaT Keratinocytes

**Figure 3:**
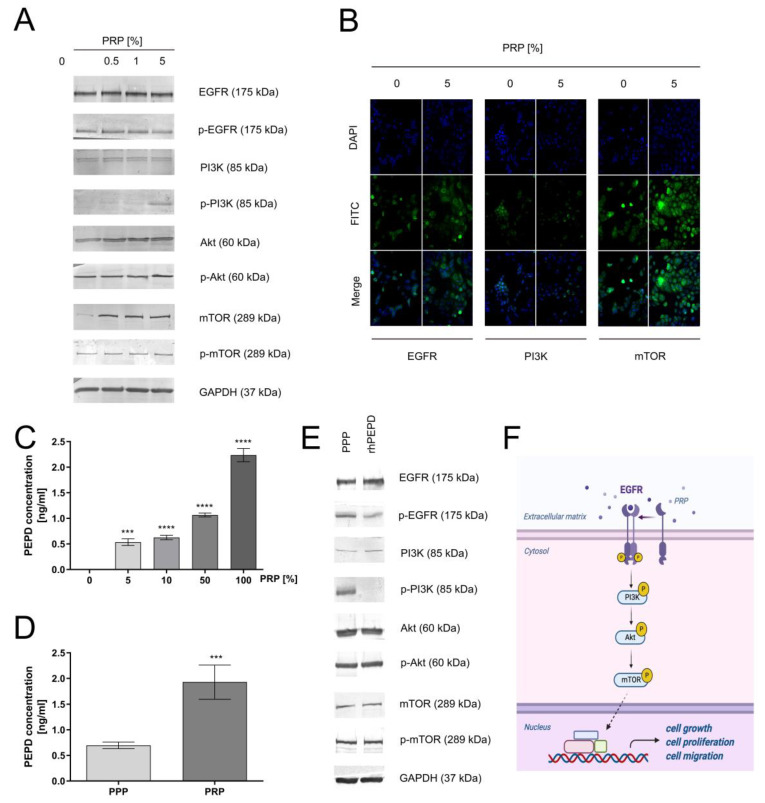
PRP-dependent activation of epidermal growth factor receptor (EGFR) and its downstream signaling proteins in HaCaT cells (**A**) PRP-mediated EGFR-downstream signaling; (**B**) Immunocytochemical staining of EGFR, PI3K and mTOR in keratinocytes treated with 5% PRP. Magnification 200×; (**C**) Prolidase (PEPD) concentration in different PRP-diluted samples measured by the ELISA kit; (**D**) PEPD concentration in platelet-poor plasma (PPP) and platelet-rich plasma measured by ELISA kit. Statistical significances were expressed using asterisks such as *** <0.001 and **** <0.0001; (**E**) Western immunoblotting of EGFR-downstream signaling in recombinant human prolidase (rhPEPD- and PPP-treated keratinocytes; (**F**) An illustration of PRP-mediated EGFR-downstream signaling. Created with BioRender.com.

To evaluate whether PRP-mediated proliferation/migration undergoes via the EGFR signaling pathway, the expressions of the receptor and its downstream proteins were measured. It is known that the PI3K/Akt/mTOR signaling pathway participates in the proliferation/migration of keratinocytes [24]. It was found that the level of total and phosphorylated forms of EGFR and PI3K/Akt/mTOR axis proteins was increased in PRP-treated keratinocytes. As can be seen in Figure 3A, PRP caused phosphorylation of EGFR (Tyr^1068^) and downstream kinases including PI3K p85 (Tyr^458^)/p55 (Tyr^199^), Akt (Ser^473^), and mTOR (Ser^2448^). The effect of PRP on keratinocytes was also evaluated using immunocytochemistry/confocal microscopy. It was observed that treatment of the cells with PRP increased the expression of EGFR, PI3K, and mTOR proteins (Figure 3B). Subsequently, we investigated whether PRP-containing prolidase is a significant player in EGFR-driven signaling since PEPD is known as an EGFR ligand [25]. Firstly, we quantified the PEPD concentration in PRP using the ELISA method. We found that PRP contains about 2 ng/mL prolidase in comparison to normal plasma (PEPD concentration below 1 ng/mL), proving that platelets are a rich source of prolidase (Figure 3C,D). Then, the expression of EGFR and downstream signaling proteins was measured in HaCaT cells cultured in prolidase-deprived plasma vs. serum-free medium containing recombinant human prolidase (rhPEPD). The presented data showed that 1 nM rhPEPD yielded similar effects on the expression of the total and active forms of EGFR, PI3K, Akt, and mTOR (Figure 3E) compared to that of poor-platelet plasma. Figure 3F illustrates a sequence of events in PRP-dependent stimulation of the EGFR-downstream signaling pathway. The results suggest that PEPD as a PRP constituent plays an important role in PRP-dependent stimulation of EGFR-downstream signaling in HaCaT cells. 

### 2.4. Activation of the β1-Integrin and IGF-1R Signaling in PRP-Treated HaCaT Cells

**Figure 4:**
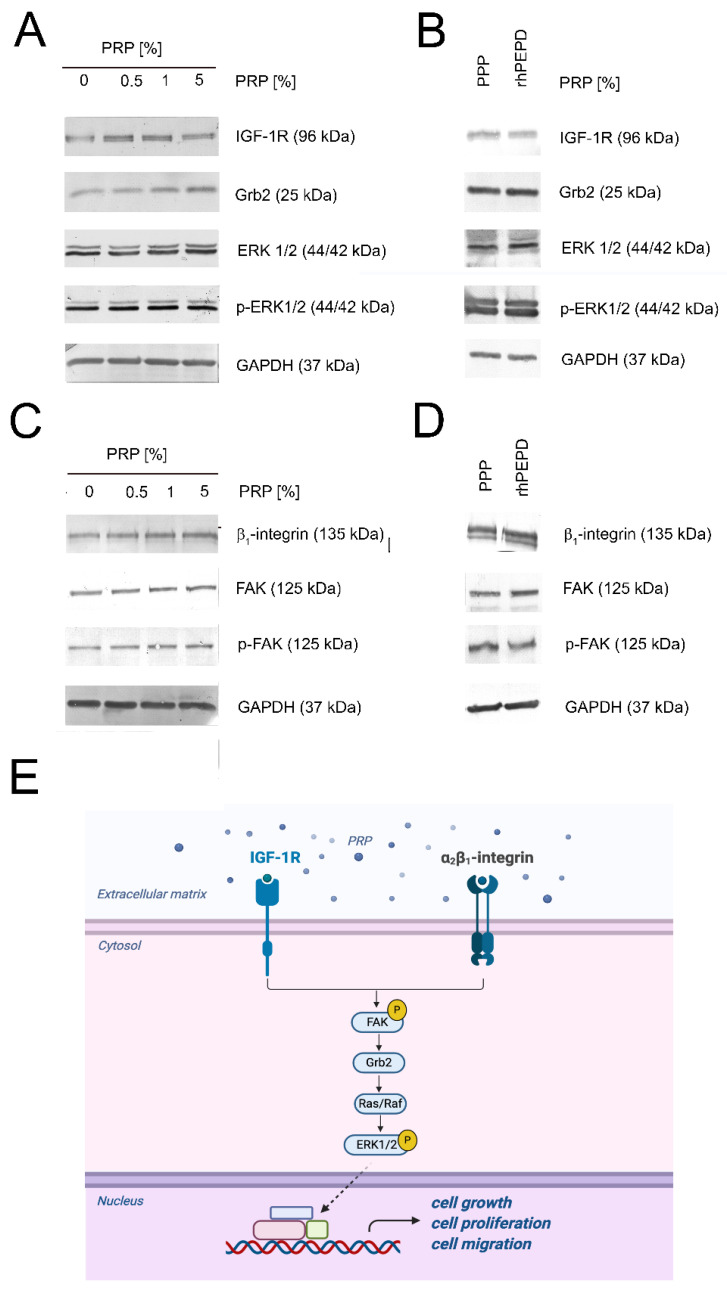
PRP-dependent activation of β_1_-integrin and IGF-1R signaling pathways in HaCaT cells (**A**) PRP-dependent activation of IGF-1R-downstream signaling pathway; (**B**) Western immunoblotting of IGF-1R-downstream signaling in rhPEPD- and PPP-treated keratinocytes; (**C**) β_1_-integrin signaling in human keratinocytes subjected to PRP; (**D**) Western immunoblotting of β_1_-integrin-downstream signaling in rhPEPD- and PPP-treated keratinocytes; (**E**) Illustration of β_1_-integrin and IGF-1R signaling pathways. Created with BioRender.com.

In PRP-treated HaCaT cells, the expressions of IGF-1R and β_1_-integrin receptor were increased. Upregulation of IGF-1R was accompanied by an increase in the expression of IGF-1R-downstream signaling proteins such as Grb2, ERK1/2, and its activated forms (phospho-ERK1/2) (Figure 4A). The level of the expression of these proteins was also assessed in rhPEPD-treated keratinocytes in comparison to PPP-treated cells. We demonstrated that this pathway was stimulated likewise in both groups (Figure 4B). Then, we investigated how PRP affected integrin receptor signaling. We also observed activation of the β_1_-integrin/FAK signaling pathway under these conditions (Figure 4C). All studied processes in PRP-treated cells were increased in a dose-dependent manner. rhPEPD at a concentration of 1 nM exerted comparable effects on β_1_-integrin-dependent signaling to that of PPP. Figure 4E illustrates down-stream signaling proteins of β_1_-integrin receptor and IGF-1R activation.

### 2.5. PRP Induces NF-κβ Expression in PRP-Treated HaCaT Cells

**Figure 5:**
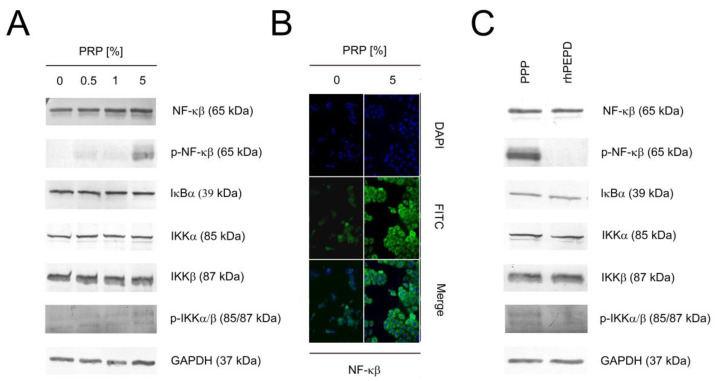
PRP induces activation of the NF-κβ signaling pathways in HaCaT cells. (**A**) PRP-dependent activation of NF-κβ signaling pathway; (**B**) Immunocytochemical staining of NF-κβ in PRP-treated keratinocytes. Magnification 200×; (**C**) Western immunoblotting of NF-κβ signaling in rhPEPD- and PPP-treated keratinocytes.

In the cytoplasm, NF-κβ is inactive. NF-κβ activation is protected by IκBα. Activation of IκBα via phosphorylation leads to ubiquitination of the inhibitory proteins and NF-κβ becomes active as a transcriptional factor [26]. In PRP-treated cells, expression of NF-κβ was increased in a dose-dependent manner as documented by western immunoblotting. We also demonstrated the phosphorylation of NF-κβ indicating activation of this transcription factor. Similarly, increases in the expression of IκBα, IKKα, and IKKβ were detected in PRP-treated cells. However, the activation of IKKα/β was slightly presented (Figure 5A). Immunocytochemical staining indicated the translocation of NF-κβ to the nucleus (Figure 5B). Later, we evaluated whether 1 nM rhPEPD activated the NF-κβ pathway and in comparison to PPP-treated cells. It has been found that both NF-κβ and IKKα/β were less stimulated by rhPEPD than prolidase-deprived plasma in keratinocytes. (Figure 5C). 

### 2.6. PRP Stimulates Collagen Biosynthesis through Enhancing Prolidase Activity and Proline Availability in An Experimental Model of Wound Healing in HaCaT Cells

**Figure 6:**
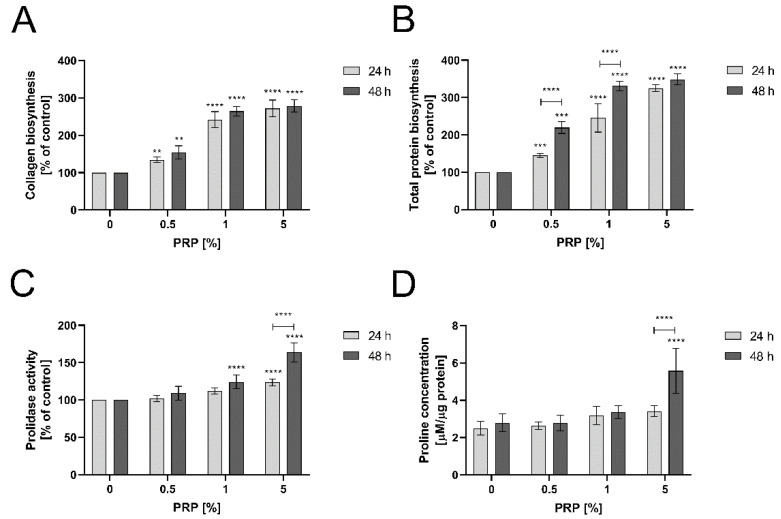
PRP stimulates collagen biosynthesis through enhancing prolidase activity and proline availability (**A**) Increased collagen biosynthesis under PRP treatment in HaCaT cells after 24 and 48 h; (**B**) Total protein biosynthesis used for normalization of collagen biosynthesis; (**C**) Prolidase activity in keratinocytes upon 24 and 48 h PRP treatment; (**D**) Proline concentration in keratinocytes treated with PRP for 24 and 48 h. Statistical significances were expressed using asterisks such as ** <0.01, *** <0.001 and **** <0.0001.

Collagen biosynthesis is required to reestablish the continuity of injured tissue [20]. The process is coordinately regulated by prolidase, which supplies proline through enzymatic hydrolysis of proline-containing dipeptides [27,28]. The role of PRP in collagen biosynthesis was studied using 5-[^3^H]-proline incorporation assay. As shown in Figure 6A, PRP stimulated collagen biosynthesis in wounded keratinocytes in a dose- and time-dependent manner. When 5% PRP was present in the culture cell medium for 24 h, collagen biosynthesis was strongly increased vs. the control (267.842 ± 27.346% of the control). Similar results were obtained after 48 h incubation with PRP. Figure 6B presents total protein biosynthesis which was used for normalization of the results of collagen biosynthesis. It shows that total protein biosynthesis is similar to collagen biosynthesis, suggesting that a predominant amount of the total protein biosynthesis is represented by collagen. Prolidase activity was also significantly enhanced upon PRP treatment. After 48 h treatment with PRP, cells exhibited enhanced activity of this enzyme (164.634 ± 18.996% of the control) (Figure 6C). An increase in prolidase activity contributed to the elevated level of free proline content in PRP-treated keratinocytes, as determined by LC-MS-based analysis. In comparison to control cells (2.797 ± 0.471 µM/µg protein), cells treated with 5% PRP increased free proline concentration up to 5.574 ± 1.216 µM/µg protein after 48 h (Figure 6D). Both increased PEPD activity and proline availability support collagen biosynthesis. 

## 3. Discussion

PRP contains multiple bioactive components including PDGF, TGF-β, VEGF, EGF, and bFGF, which have fundamental roles in wound healing. These factors are known to regulate processes such as cell migration, proliferation and differentiation, chemotaxis and to promote extracellular matrix (ECM) accumulation by binding to specific cell surface receptors [29]. As PRP is rich in these growth factors, PRP may serve as a promising agent for an acceleration of the wound healing process. This process is complex and includes many cell types. Among them are keratinocytes which play a significant role in the epithelization phase [20]. Since epithelial cell migration is commonly observed in both chronic and acute skin disturbances, promoting this process enhances healing the wound. 

In this study, we attempted to investigate the effect of PRP on selected biological processes in human keratinocytes in vitro including cell migration, proliferation, collagen metabolism, and cell cycle regulation. Moreover, we evaluated the expression of EGFR, β_1_-integrin, and IGF-1R-dependent signaling proteins. Since PRP contains prolidase [1], we hypothesized that PRP-dependent functions (in the context of wound healing) could be at least in part due to prolidase. It is known that PEPD is a ligand of EGFR and activates its downstream signaling proteins [25]. Therefore, the interest was focused on the significance of prolidase in the biological effects of PRP. Our data indicated that even at low concentration (0.5%) PRP augmented cell migration and proliferation. It is well established that EGFR-mediated signaling results in the promotion of cellular growth, migration, and proliferation. We observed that PRP-treated HaCaT cells upregulated the expression of EGFR and downstream proteins such as PI3K, Akt, and mTOR. Moreover, strong activation of β_1_-integrin and IGF-1 receptors was observed. All the above-mentioned signaling pathways regulate cellular processes facilitating wound healing and restoring tissue integrity. It is of great importance in the repair process since the role of both receptors in anabolic processes is well established [1]. Their role is particularly important in collagen biosynthesis. Both β_1_-integrin and IGF-1 receptors transmit signals that induce collagen biosynthesis [30,31]. Intracellular prolidase activity was also increased, supporting proline for collagen biosynthesis. This process is of critical importance in the last step of wound healing and scar formation [1]. Collagen biosynthesis relates to β_1_-integrin signaling leading to activation of FAK that interacts with Grb2 and then through Sos, Ras, and Raf proteins signal reaches ERK1/2 [32,33]. The end-point event of this cascade is the induction of gene transcription promoting the expression of genes of the key proteins regulating cell growth, differentiation, and metabolism [34]. The analysis of expressions of these signaling proteins confirmed the mechanism of PRP-induced anabolic processes.

We also addressed the question of how PRP affected the cell cycle of HaCaT cells. We found that PRP exerts cell cycle progression in keratinocytes via regulating phase G1, S, and G2/M. The entry of eukaryotic cells into mitosis is strictly regulated at several steps including cyclin B1 and phosphorylation of cdc2. Then, once the cell overcomes the G1/S checkpoint to start DNA synthesis, cyclin E is expressed, while cyclin A is required for both S-phase and M-phase. We suggest that PRP-dependent up-regulation of expressions of cell cycle regulatory proteins explains the mechanism for stimulation of the cell proliferation. Our results correspond to the study conducted by Kim et al. [35] who presented upregulation of cyclin D and CDK in PRP-treated HaCaT cells. Thus, the activation of cyclins by PRP may influence the epidermal cells to promote the wound healing process. 

Our results also demonstrated that NF-κβ expression was upregulated in PRP-treated HaCaT cells, indicating a link to the inflammatory phase of wound healing. An increased NF-κβ-mediated inflammatory response (COX-2, iNOS, and NO) may facilitate cell proliferation [36]. NO, as an inflammatory cytokine, has been demonstrated to induce VEGF production in macrophages [37]. It is also indicated that the cells involved in the healing process may release cytokines and growth factors that act as paracrine factors for VEGF induction. In vivo increased expression of VEGF indicates the triggering of angiogenesis to re-establish the capillary network and provides nutrients to newly proliferative cells. NF-κβ and PI3K signaling is involved in the induction of VEGF [38]. Moreover, an increased nuclear HIF-1α level and elevated expression of HIF-1α-dependent gene products, VEGF, and glucose transporter-1 (Glut-1) [24,27,39] are involved in angiogenesis (among four tissue regeneration steps). It is well established that PRP facilitates the tissue repair process. Due to the high content of prolidase and growth factors contained in PRP, it has been widely used in regenerative medicine, especially in acute and chronic soft tissue injuries [1,40,41,42,43]. 

Furthermore, one of the important points highlighted by the present study is the potential role of prolidase as a ligand of EGFR in an acceleration of wound healing. These studies provide a basis for further studies on the role of prolidase in the mechanisms of PRP-dependent wound healing.

## 4. Materials and Methods 

### 4.1. Platelet-Rich Plasma Production

Platelet-rich plasma was obtained as we previously described [1]. The concentration of PLTs, WBCs, and RBCs for PRP and VB (venous blood) was presented in the Supplementary Materials (Tables S1 and S2). The ethics committee of the Medical University of Bialystok approved the study (R-I-002/33/2015, 29 Jan 2015) and all participants provided written informed consent before enrollment.

### 4.2. HaCaT Cell Culture and Treatment

Spontaneously immortalized HaCaT cell line purchased from Cell Line Service (Eppelheim, Germany) was grown in DMEM cell culture medium (PAN-Biotech, Aidenbach, Bavaria, Germany) containing 10% fetal bovine serum (Gibco, Carlsbad, CA, USA) and 1% Penicillin/Streptomycin (Gibco, Carlsbad, CA, USA) at 37 °C in a humidified atmosphere of 5% CO_2_. For experiments, cells (4–7 passages) were treated with PRP at the concentrations of 0.05–5% for 30 min, 24 or 48 h, platelet-poor plasma at the concentrations of 5% for 30 min and 24h, and recombinant human prolidase (purity >95% SDS-PAGE; Abcam, Cambridge, United Kingdom) at the concentration of 1 nM for 30 min and 24 h.

### 4.3. DNA Biosynthesis Assay

DNA biosynthesis of HaCaT cells was evaluated with the CyQUANT^®^ Cell Proliferation Assay (Thermo Fisher Scientific, Waltham, MA, USA). PRP-treated HaCaT cells (0.05–5% for 24 and 48 h) were washed twice with PBS (pH 7.4) and frozen at −80 °C until the day of analysis. The assay was performed according to the manufacturer’s protocol. Fluorescence was read on the TECAN Infinite^®^ M200 PRO (Tecan Group Ltd., Männedorf, Switzerland) with filters set at 480/520 nM. 

### 4.4. Wound Healing Assay

Confluent keratinocytes were scratched with a sterile 200 μL pipette tip, washed with PBS 2 times, and supplemented with PRP (0.5–5% for 24, and 48 h). An inverted optical microscope (Nikon, Minato, Tokyo, Japan) with a 40× magnification was employed to capture images of the injury area. The wound closure rate was counted by ImageJ software (https://imagej.nih.gov/ij/, National Institutes of Health, Bethesda, MD, USA) and calculated as previously described [13]. 

### 4.5. Cell Cycle Analysis

HaCaT cells were PRP-treated (0.5–5%) for 24 h. After incubation, cells were trypsinized, centrifuged (5 min, 500× *g*), and washed twice with PBS. The pellet was suspended in 0.5 mL of PBS, fixed in 4.5 mL of ice-cold 70% ethanol, and stored at 4 °C until analysis. On the day of analysis, ethanol-fixed cells were centrifuged (5 min, 500× *g*), washed in PBS, and then suspended in Solution 3 (ChemoMetec, Allerod, Denmark) followed by incubation at 37 °C for 5 min. The fixed cell cycle analysis was conducted using an image cytometer (NC-3000, ChemoMetec, Allerod, Denmark).

### 4.6. Preparation of Cell Lysates

Cells were cultured on a 100 mm dish in FBS-free DMEM with PRP (0.5–5%) for 30 min and 24 h. Then, cells were collected as previously described [13]. Protein concentration was measured using the Pierce BCA assay kit (Thermo Fisher Scientific, Waltham, MA, USA). Cell lysates were subjected to western immunoblotting and prolidase activity measurement. 

### 4.7. Western Immunoblotting 

Equal amounts of protein samples were separated on SDS-PAGE gels (7.5–10%) and electro-transferred onto polyvinylidene difluoride membranes (BioRad Laboratories, Hercules, CA, USA) which blocked with 5% non-fat dried milk (Santa Cruz Biotechnology, Dallas, TX, USA) or bovine serum albumin (Sigma Aldrich, Saint Louis, MO, USA) in TBS-T (20 mM Tris, 150 mM NaCl, 0.1% Tween-20, pH 7.6) for 1 h at room temperature on a rotator. Then, the membranes were incubated with primary antibodies overnight at 4 °C, followed by incubation with alkaline phosphatase-linked goat anti-rabbit or anti-mouse antibodies for 1h at RT. The list of primary and secondary antibodies is enclosed as Supplementary Materials (Tables S3 and S4). The bands were visualized using 1-Step™ NBT/BCIP Substrate Solution (Thermo Fisher Scientific, Waltham, MA, USA) and their intensities were semi-quantitatively calculated with ImageJ software (https://imagej.nih.gov/ij/, National Institutes of Health, Bethesda, MD, USA). All experiments were repeated thrice. 

### 4.8. Confocal Microscopy (Immunocytochemistry)

After 24 incubation with 5% PRP, cells were treated as previously described [13]. A confocal laser scanning microscope (BD Pathway 855 Bioimager, Becton Dickinson, Franklin Lakes, NJ, USA) with AttoVision software was used for image visualization.

### 4.9. Prolidase Activity Measurement

The activity of prolidase was determined according to the method published by Besio et al. [44]. Absorbance was read at 515 nM on TECAN Infinite^®^ M200 PRO (Tecan Group Ltd., Männedorf, Switzerland). 

### 4.10. Determination of Collagen Biosynthesis

Collagen biosynthesis was evaluated by incorporation of 5-[^3^H]-proline (5 μCi/mL; Hartmann Analytic, Germany) into proteins prone to bacterial *Clostridium histolyticum* collagenase (Sigma Aldrich, Saint Louis, MO, USA) according to Peterkofsky’s method [45]. Radiometric measurement was run on the Liquid Scintillation Analyzer Tri-Carb 2810 TR (PerkinElmer, Waltham, MA, USA). Total protein biosynthesis served as a normalization. 

### 4.11. Prolidase Concentration Measurement by ELISA

Prolidase concentration in platelet-poor and platelet-rich plasma samples was measured using an ELISA kit (Wuhan, Hubei, China). The assay was performed accordingly to the instructions provided by the manufacturer. The prolidase concentration test was run at least in triplicates. 

### 4.12. LC-MS Analysis of Proline Concentration

Proline concentration in culture HaCaT cells was measured with the use of the method published by Klupczynska et al. [46]. Samples were analyzed using Agilent 1260 Infinity HPLC system coupled to Agilent 6530 Q-TOF mass spectrometry detector (Agilent Technologies, Santa Clara, CA, USA) with electrospray ionization as an ion source in positive ionization mode. Samples were injected onto a HILIC column (Luna HILIC, 2 × 100 mm, 3 µm, Phenomenex, Torrance, CA, USA). Methanol—extracted cell lysates were collected in triplicates and injected in duplicates. Total protein concentration was used for normalization and presented as µM/µg protein.

### 4.13. Statistical Analysis

All experiments were carried out at least in three replicates and the experiments were repeated at least three times. Data are shown as a mean ± standard error (SEM). For statistical analysis, ANOVA with Dunnett’s correction and *t*-test were used and performed using GraphPad Prism 5.01 (GraphPad Software, San Diego, CA, USA). Statistical significances were expressed using asterisks such as * <0.05, ** <0.01, *** <0.001 and **** < 0.0001.

## 5. Conclusions

The data suggest that in a model of wound healing in keratinocytes, PRP induces complex growth factors and adhesion receptor-dependent signaling that stimulate cell proliferation, migration, and collagen biosynthesis. Since PRP induces a strong EGFR signal in this process and prolidase as a ligand of EGFR is present in PRP it indicates that prolidase could play important role in PRP-induced wound healing in keratinocytes. These studies provide a basis for further studies on the mechanisms of PRP-dependent wound healing.

## Data Availability

The datasets used and/or analyzed during the current study are available from the corresponding author on reasonable request.

## Supplementary Materials

Supplementary materials can be found at https://www.mdpi.com/1422-0067/22/2/936/s1. Figure S1. Cell viability of PRP-treated HaCaT cells for 24–72 h. Table S1. Basic blood parameters measured in whole blood. Table S2. Basic blood parameters measured in platelet-rich plasma (PRP) fraction. Table S3. The list of primary antibodies used in Western blot and immunocytochemistry. Table S4. The list of secondary antibodies used in Western blot and immunocytochemistry.

## Abbreviations

Akt	protein kinase B
EGFR	epidermal growth factor receptor
ERK1/2	extracellular signal-regulated kinase 1/2
FAK	focal adhesion kinase pp125FAK
GAPDH	glyceraldehyde 3-phosphate dehydrogenase
Glut-1	glucose transporter-1
Grb2	growth factor receptor-bound protein 2
HIF-1α	hypoxia-inducible factor 1 alpha
IGF-1R	insulin-like growth factor 1 receptor
mTOR	mammalian target of rapamycin
NF-κβ	nuclear factor kappa beta
IκBα	inhibitor of nuclear factor-kappa beta
IκKα	IκB kinase alpha
IκKβ	IκB kinase beta
PEPD	prolidase
PI3K	phosphoinositide 3 kinase
PRP	platelet-rich plasma
PPP	platelet-poor plasma
TGF-β1	transforming growth factor-beta 1
VEGF	vascular endothelial growth factor
