# Supplementary material for: Platelet-Rich Plasma Promotes the Proliferation of Human Keratinocytes via a Progression of the Cell Cycle. A Role of Prolidase
Source: Int J Mol Sci. 2021 Jan 19;22(2):936. doi: 10.3390/ijms22020936 (PMC7832888; doi:10.3390/ijms22020936)
Supplement: Supplementary file 1 [file ijms-22-00936-s001.pdf]

Supplementary Materials

Article

Platelet-Rich Plasma Promotes the Proliferation of Human Keratinocytes via a Progression of the Cell Cycle. A Role of Prolidase

Magdalena Misiura [1], Tomasz Guszczyn [2], Ilona Oscilowska [3], Weronika Baszanowska [3], Jerzy Palka [3] and Wojciech Miltyk [1,*]

[1] Department of Analysis and Bioanalysis of Medicines, Medical University of Bialystok, Kilińskiego 1, 15-089 Bialystok, Poland; magdalena.misiura@umb.edu.pl

[2] Department of Pediatric Orthopaedics and Traumatology, Medical University of Bialystok, Kilińskiego 1, 15-089 Bialystok, Poland; tombial@me.com

[3] Department of Medicinal Chemistry, Medical University of Bialystok, Kilińskiego 1, 15-089 Bialystok, Poland; ilona.zareba@gmail.com (I.O.); w.baszanowska22@wp.pl (W.B.); pal@umb.edu.pl (J.P.)

* Correspondence: wojciech.miltyk@umb.edu.pl; Tel.: +48-85-748-5845

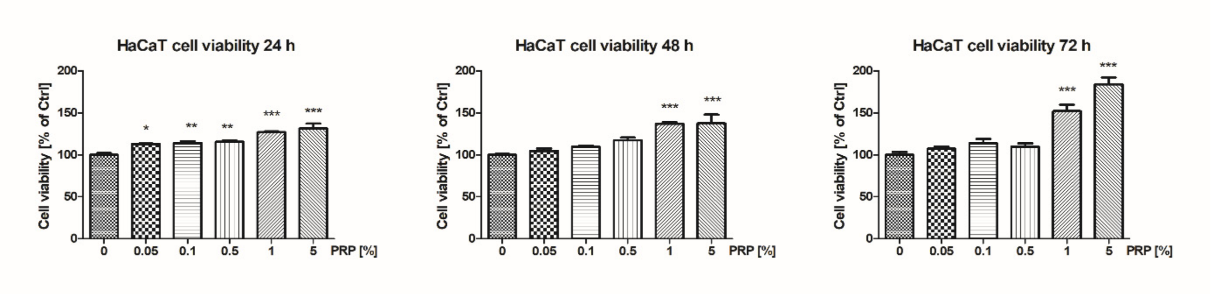

Figure S1. Cell viability of PRP-treated HaCaT cells for 24-72 h. Statistical significances were expressed using asterisks such as * < 0.05, ** < 0.01, *** < 0.001 and **** < 0.0001.

Table S1. Basic blood parameters measured in whole blood.

Parameter	Unit	Average	Standard deviation
WBC	[10´3/µl]	6,13	1,59
RBC	[10´6/µl]	4,92	0,49
HGB	[g/dl]	13,85	1,12
HCT	[%]	39,95	5,40
MCV	[fl]	83,42	3,83
MCH	[pg]	28,19	1,24
MCHC	[g/dl]	48,83	67,13
PLT	[10´3/µl]	259,25	49,31
RDW-SD	[fl]	39,00	1,70
RDW-CV	[%]	13,03	0,41
MPV	[fl]	10,44	0,76
PCT	[%]	0,27	0,04
PDW	[fl]	12,29	1,49
P-LCR	[%]	26,94	7,68

Table S2. Basic blood parameters measured in platelet-rich plasma (PRP) fraction.

Parameter	Unit	Average	Standard deviation
WBC	[10´3/µl]	9,04	6,48
RBC	[10´6/µl]	1,99	2,52
HGB	[g/dl]	5,49	6,70
HCT	[%]	16,54	19,50
MCV	[fl]	87,47	5,62
MCH	[pg]	28,05	1,34
MCHC	[g/dl]	32,14	1,94
PLT	[10´3/µl]	906,45	474,07
RDW-SD	[fl]	37,74	2,23
RDW-CV	[%]	12,99	1,40
MPV	[fl]	9,98	0,78
PCT	[%]	0,90	0,48
PDW	[fl]	11,28	1,52
P-LCR	[%]	24,76	5,97

Table S3. The list of primary antibodies used in Western blot and immunocytochemistry.

**Primary antibodies**	**Dilution**	**Vendor**	**Techniques**
p44/42 MAPK (ERK1/2) Rabbit mAb	1:1000	Cell Signaling Technology	Western blot
mTOR Rabbit mAb	1:1000	Cell Signaling Technology	Western blot
EGF Receptor Rabbit mAb	1:1000	Cell Signaling Technology	Western blot
IGF-1 Receptor β Rabbit mAb	1:1000	Cell Signaling Technology	Western blot
Phospho-EGF Receptor (Tyr1068) Rabbit mAb	1:1000	Cell Signaling Technology	Western blot
Phospho-p44/42 MAPK (ERK1/2) Rabbit mAb	1:1000	Cell Signaling Technology	Western blot
FAK Rabbit mAb	1:1000	Cell Signaling Technology	Western blot
Phospho-FAK (Tyr397) Rabbit mAb	1:1000	Cell Signaling Technology	Western blot
Integrin β_1 Receptor Rabbit mAb	1:2000	Cell Signaling Technology	Western blot
Akt Rabbit mAb	1:2000	Cell Signaling Technology	Western blot
Phospho-Akt (Ser473) Rabbit mAb	1:1000	Cell Signaling Technology	Western blot
Phospho-mTOR (Ser2448) Rabbit mAb	1:1000	Cell Signaling Technology	Western blot
PI3 Kinase p85 Rabbit mAb	1:1000	Cell Signaling Technology	Western blot
Phospho-PI3 Kinase p85 (Tyr458)/p55 (Tyr199) Antibody	1:1000	Cell Signaling Technology	Western blot
NF-κβ p65 Rabbit Antibody	1:1000	Cell Signaling Technology	Western blot
GAPDH Rabbit mAb	1:1000	Cell Signaling Technology	Western blot
Phospho-cdc2 (Tyr15) (10A11) Rabbit mAb	1:1000	Cell Signaling Technology	Western blot
Cyclin E1 (D7T3U) Rabbit mAb	1:1000	Cell Signaling Technology	Western blot
Cyclin B1 (D5C10) XP® Rabbit mAb	1:1000	Cell Signaling Technology	Western blot
Thymidine Kinase 1 (E2H7Z) Rabbit mAb	1:1000	Cell Signaling Technology	Western blot
Cyclin A2 (E1D9T) Rabbit mAb	1:1000	Cell Signaling Technology	Western blot
CDT1 (D10F11) Rabbit mAb	1:1000	Cell Signaling Technology	Western blot
IKKα (3G12) Mouse mAb	1:1000	Cell Signaling Technology	Western blot
IKKβ (D30C6) Rabbit mAb	1:1000	Cell Signaling Technology	Western blot
Phospho-IKKα/β (Ser176/180) (16A6) Rabbit mAb	1:1000	Cell Signaling Technology	Western blot
Phospho-NF-κB p65 (Ser536) (93H1) Rabbit mAb	1:1000	Cell Signaling Technology	Western blot
Phospho-IκBα (Ser32) (14D4) Rabbit mAb	1:1000	Cell Signaling Technology	Western blot
Grb2 Mouse Antibody	1:500	Becton Dickin	Western blot
EGF Receptor Rabbit mAb	1:500	Cell Signaling Technology	Immunoflourescence
PI3 Kinase p85 Rabbit mAb	1:100	Cell Signaling Technology	Immunoflourescence
mTOR Rabbit mAb	1:100	Cell Signaling Technology	Immunoflourescence

Table S4. The list of secondary antibodies used in Western blot and immunocytochemistry.

Secondary antibodies	Dilution	Vendor	Techniques
Secondary AP-conjugated anti-mouse Antibody	1:10000	Sigma Aldrich	Western blot
Secondary AP-conjugated anti-rabbit Antibody	1:10000	Cell Signaling Technology	Western blot
Secondary FITC-conjugated anti-rabbit Antibody	1:500	Becton Dickinson	Immunoflourescence
